# Effect of Probiotics on Uric Acid Levels: Meta-Analysis with Subgroup Analysis and Meta-Regression

**DOI:** 10.3390/nu17152467

**Published:** 2025-07-29

**Authors:** Rym Ben Othman, Mouna Ben Sassi, Syrine Ben Hammamia, Chadli Dziri, Youssef Zanina, Kamel Ben Salem, Henda Jamoussi

**Affiliations:** 1 Institut National de Nutrition et de Technologie Alimentaire de Tunis, 11 Rue Jbal Lakhdhar, Tunis 1007, Tunisia; hendajamoussi@gmail.com; 2 Faculty of Medicine of Tunis, University of Tunis El Manar, 13 Rue Jbal Lakhdhar, Tunis 1007, Tunisia; mouna.bs@hotmail.fr (M.B.S.); syrine.benhammamia@fmt.utm.tn (S.B.H.); 3 National Centre Chalbi Belkahia of Pharmacovigilance, Department of Clinical Pharmacology, 9, Avenue Dr. Zouhaier ESSAFI, Tunis 1006, Tunisia; 4 Department of Clinical Pharmacology, Research Laboratory of Clinical and Experimental Pharmacology (LR16SP02), Tunis El Manar University, Tunis 1006, Tunisia; 5 General Surgery Department B of General Surgery, University of Tunis el Manar, Boulevard 9 Avril 1938, Tunis 1006, Tunisia; chadli.dziri@planet.tn; 6 Department of Preventive Medicine, University of Medicine of Monastir, Avenue Avicenne, Monastir 5019, Tunisia; zanina.youssef@protonmail.com (Y.Z.); kbsalem@gmail.com (K.B.S.); 7 Research Unit of Obesity: Etiopathogenesis, Pathophysiology and Treatment Research Unit (UR18ES01), National Institute of Nutrition and Food Technology, 11 Rue Jebel Lakhdar 1007, BebSaadoun, Tunis 1007, Tunisia

**Keywords:** uric acid, probiotics, microbiota, metabolic disease, gout, randomized trials, meta-analysis, subgroup analysis, meta-regression

## Abstract

Background: Probiotics can modulate the microbiota and decrease uric acid levels. Objectives: This meta-analysis aimed to assess the effects of probiotics on uric acid levels. Methods: The keywords “probiotics”, “uric acid”, “gout”, “hyperuricemia” were searched in PubMed Medline, EMBASE, Web of Science, and Google Scholar. The search was limited to the English, French, Italian, and Spanish languages, and to the period between 1 January 2000 to 30 August 2024. We included RCTs and observational studies comparing probiotics to placebo. We excluded studies reporting (1) prebiotics, symbiotics, or postbiotics, (2) animal studies, and (3) case reports, commentaries, or reviews. Two independent reviewers performed quality assessment and data extraction. This meta-analysis was performed according to the PRISMA 2020 and AMSTAR 2 guidelines. The main outcome measure was uric acid levels “after–before” probiotic versus placebo interventions. Forest plots summarized the data using a random model. Results: Nine studies included 394 patients, of whom 201 were treated with probiotics and 193 with placebo. There was a statistically significant difference in favor of the probiotic group compared with the control group regarding the main outcome measure. However, substantial heterogeneity was noted, explained (after applying subgroup analysis and meta-regression) by the following moderators: continent, diseased/healthy, male sex, and monostrain probiotics. Conclusions: This meta-analysis demonstrates that probiotics reduced uric acid levels in Asian males who had disease and were treated with monostrain probiotics.

## 1. Introduction

Hyperuricemia (HUA) is a metabolic disease, and its main pathogenesis is purine metabolism disorder [1]. An increase in uric acid levels, known as HUA, poses significant health risks, particularly in relation to cardiovascular disease (CVD) and gout. A population-based survey in 2011 revealed that the morbidity of gout and asymptomatic HUA in the United States was 3.9% and 21.4%, respectively [2]. This condition is becoming increasingly prevalent in various populations and is influenced by lifestyle, eating habits, and genetic factors. Elevated uric acid is associated with hypertension, arterial stiffness, and heart failure, serving as an independent risk factor for CVD [3]. HUA is also a major contributor to gout, characterized by painful joint inflammation due to urate crystal deposition [4].

The incidence of HUA is increasing worldwide [5], in correlation with the increasing rates of CVD. From 2005 to 2006, the number of patients with HUA in the USA reached 47.1 million, and the overall prevalence was reported to be 20.1% [1]. Another study indicated that the prevalence of HUA ranges from 11.3% to 47% in the USA, 11.9% to 25.0% in Europe, and 13.1% to 13.3% in China [2].

Conventional treatments for HUA, while beneficial to some extent, face significant challenges that can limit their effectiveness. Traditional pharmacological approaches such as xanthine oxidase inhibitors and uricosuric agents have shown mixed results, particularly in patients with chronic kidney disease (CKD) where treatment may not alter disease progression [3]. Furthermore, the efficacy of these treatments may be inconsistent due to the multifactorial nature of HUA [4,5]. Conventional medications like allopurinol, colchicine and febuxostat may not provide consistent results across different patient populations [6]. On the other hand, these therapies can cause adverse effects, limiting their long-term use and patient adherence [7,8].

Probiotics have emerged as a promising approach for managing uric acid (UA) levels, particularly in the context of HUA since 2022 [9]. Recent studies indicate that specific probiotic strains can modulate urate metabolism, reduce inflammatory responses, and improve metabolic parameters, thereby contributing to UA management [10,11]. Probiotics such as *Lactobacillus johnsonii* YH1136 inhibit hepatic xanthine oxidase (XOD) activity, a key enzyme in UA production, thus lowering serum UA levels [10]. A clinical trial demonstrated that a combination of *Lactobacillus* strains significantly reduced serum UA levels and improved liver function in participants with metabolic-associated fatty liver disease [12]. Research in humans is still scarce, and probiotics are not cited in guidelines for the management of hyperuricemia (HUA). In light of these facts, this meta-analysis aimed to assess the effect of probiotics supplementation (PBs) versus placebo on uric acid levels.

## 2. Materials and Methods

This systematic review with meta-analysis was performed according to the PRISMA guidelines 2020 [13]. The protocol has been registered in PROSPERO with the ID CRD42024527159.

### 2.1. Electronics Searches

Only human studies were considered. The references list of identified articles was also checked to identify further studies. Healthy patients and those with any diseases with values of uric acid reported were considered for inclusion. Supplementation with prebiotics or symbiotics were excluded. The quality of the studies was evaluated by two authors (RBO and MBS). In case of discordance, discussion with CD was carried out.

We performed an electronic investigation of the relevant literature published during the past two decades, from 1 January 2000 to 30 August 2024. Language restrictions were applied, limited to English, French, Arabic, Spanish, and Italian. We searched in the following databases: Scopus, Web of Science, National Institutes of Health PubMed/MEDLINE, and Google Scholar. This research was carried out manually and using MeSH. We used the following keywords: “probiotics”, “uric acid”, “gout”, and “hyperuricemia”.

### 2.2. Eligibility (Inclusion and Exclusion) Criteria

We retained RCTs and non-randomized clinical trials (CCTs) comparing outcomes after probiotic versus placebo supplementation. Data from review articles, editorials letters, abstracts, comments, books, protocols, congress reports, guidelines, and case series (fewer than ten cases) were excluded. When data were missing, we sent the authors an e-mail about missing primary outcome information, if we did not receive a response, the study was excluded.

### 2.3. Primary Outcome Measure

The primary outcome measure was “the variation of the uric acid value (as a continuous variable) before and after intervention: probiotic versus placebo”.

### 2.4. Data Collection and Analysis

Two authors (RBO and MBS) independently reviewed all abstracts. Randomized con-trolled trials (RCTs) and clinical controlled trials (CCTs) were considered. The full texts of all studies that met the inclusion criteria were retrieved. All studies that met the selection criteria were independently appraised by two authors according to the Jadad Statement for RCTs and MINORS for CCTs [14]. RBO and MBS independently extracted the data from the retained studies. Disparities were settled after discussion with a third author (CD). The studies were fully matched in terms of the study period, journal, year of publication, number of patients, their age and sex, diseases, and BMI.

If studies presented the results as the median and interquartile range (IQR) or range, we converted the values to mean and SD according to the Cochrane handbook 7.7.3.5. if the authors didn’t mention the deviation standard (SD) but only the confidence interval, we imputed the corresponding SD according to formula provided by Cochrane Handbook [15].

### 2.5. Evaluation of Effect Size

According to the primary outcome “the variation of the uric acid value before and after intervention: probiotic versus placebo” for each study (which is a continuous variable), we extracted the uric acid value before probiotic treatment, called “T probiotic 0”, and after probiotic treatment, called “T probiotic1”, for each study. We did the same exercise for the placebo group: “T placebo 0” before placebo and “T placebo 1” after placebo. We calculated the difference in means for the “after–before” probiotic and “after–before” placebo groups for each study using Cohen’s d index, as the standard difference in means [difference in mean outcome before and after for the two groups/standard deviation of outcome among participants]. Then, we obtained the standard difference in means (Std diff in means) for the probiotic group and placebo group in each study.

Afterwards, another comparison between the probiotic and placebo groups was performed for each study, again using Cohen’s d index to calculate the mean effect size. Forest plots summarized the data using a random model.

Publication bias was evaluated using a funnel plot followed by Duval and Tweedie’s trim and fill method, and sensitivity analysis was performed using the “one study removed” method.

For assessment of heterogeneity, we calculated the Cochrane Chi^2^ test (Q-test), variance Tau^2^, and 95% predictive interval (PI) [16,17,18].

Reasons for heterogeneity were investigated by testing interactions between relevant factors, termed moderators (age, gender, mono strain/mix, healthy or diseased subject, country of origin (continent), duration of supplementation, body mass index, median follow-up), and effect size. Meta-regression was performed for continuous variables [19] and subgroup analysis for dichotomic variables. Comprehensive meta-analysis software version 4 was used for all calculations [20]. The level of significance was 0.05 for all calculations except for the Q test *p* ≤ 0.1.

## 3. Results

### Retrieved Studies

**Figure 1:**
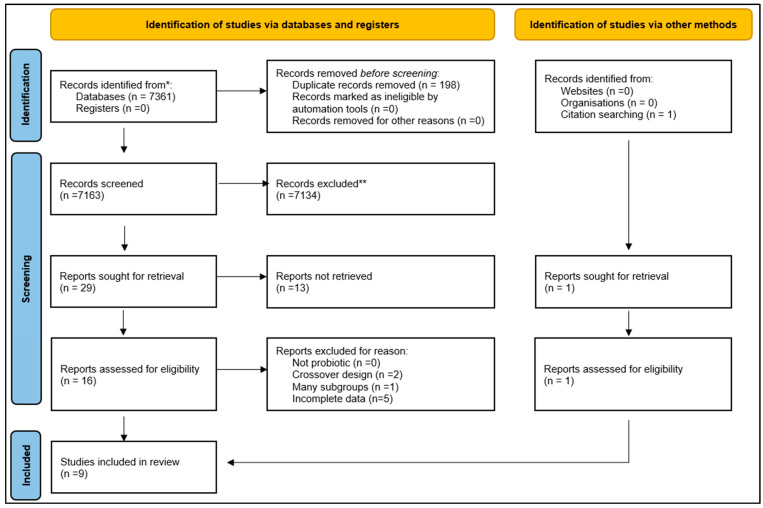
Flowchart of included studies. * Consider, if feasible to do so, reporting the number of records identified from each database or register searched (rather than the total number across all databases/registers). ** If automation tools were used, indicate how many records were excluded by a human and how many were excluded by automation tools [13]. This work is licensed under CC BY 4.0. To view a copy of this license, visit https://creativecommons.org/licenses/by/4.0/ (accessed on 23 March 2025).

In total, 7361 articles were identified (Figure 1). One article was found from citation searching. Nine studies [9,21,22,23,24,25,26,27,28] published from 1 January 2000 to 30 August 2024 met the eligibility criteria (Figure 1). Szulinska [26] was divided in two studies because they referred to groups with the same probiotics at different doses (low dose Szulinska (a) and high dose Szulinska (b)). Eight studies were RCTs. Ben Othman’s study [21] was a prospective comparative non-randomized study. These studies involved 394 patients. Two studies were from Iran [22,24], one study was from Tunisia [21], another from Serbia [23], and another from Poland [26]. The study by Rodriguez and al was from Spain [25] and was another from Austria [28]. Yamanaka et al. reported from Japan [27] and Zhao [9] from China (Table 1).

Of the eight RCTs included in the review, one study was assigned “low quality” because it was not double-blind. The MINORS score of the non-randomized study by Ben Othman [21] was 18/24.

**Figure 2:**
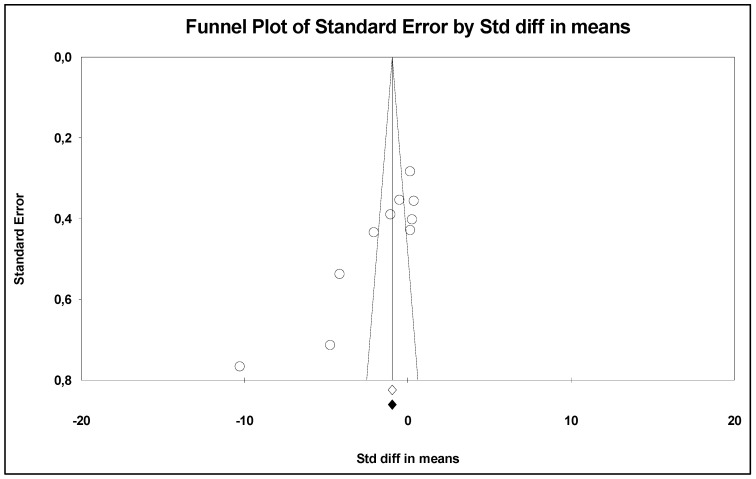
Funnel plot showing no publication bias.

A total of 394 patients were included, of whom 201 were in probiotics and 193 in placebo groups. Sex ratio (M/W) varied between 0 to 22. The ages of the included patients ranged from 22 to 63 years old. All patients included had a disease except in Michalichova and Fabian’s studies (Table 1). The funnel plot revealed no publication bias (Figure 2).

The main outcome measure was uric acid levels “after–before” probiotic versus placebo interventions.

**Figure 3:**
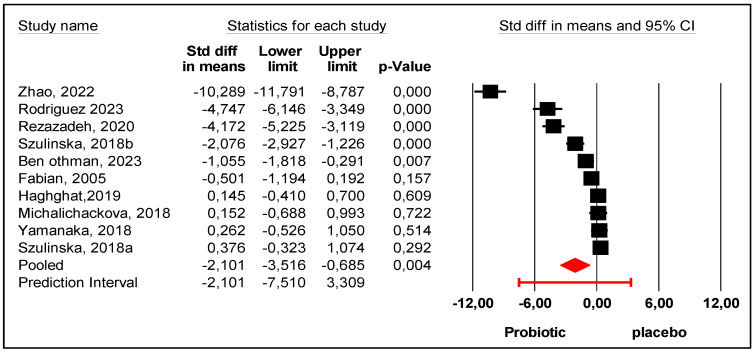
Forest plot of uric acid levels “after-before” probiotic versus placebo interventions [9,21,22,23,24,25,26,27,28].

One study [27] reported an increase in UA levels after probiotic intervention. Five reported decreases, but the differences were not significant [21,23,26,28], and four reported significant decreases in UA levels [9,22,24,25]. The mean effect size (Figure 3), represented by the Cohen d index was equal to −2.101 with 95% CI (−3.516 to −0.685), confirming that probiotics significantly decreased uric acid levels compared with placebo. If we consider the mean effect size of −2.101 as the true effect size, the 95% prediction interval was between (−7.510 and +3.309) which showed substantial heterogeneity. To explain this heterogeneity, we looked for moderators such as age, gender, multiple strains (mix) or a single strain (mono), continent, study period, initial population (healthy or diseased), and BMI. We also searched for studies on treatments for lowering uric acid; however, only one study reported the simultaneous intake of colchicine and allopurinol [25], which was insufficient to include as a moderator in the analysis.

We applied subgroup analyses for moderators including continent, patient health status at inclusion, and mono or multiple strains, and meta-regression for age, gender, BMI, and median follow-up.

**Figure 4:**
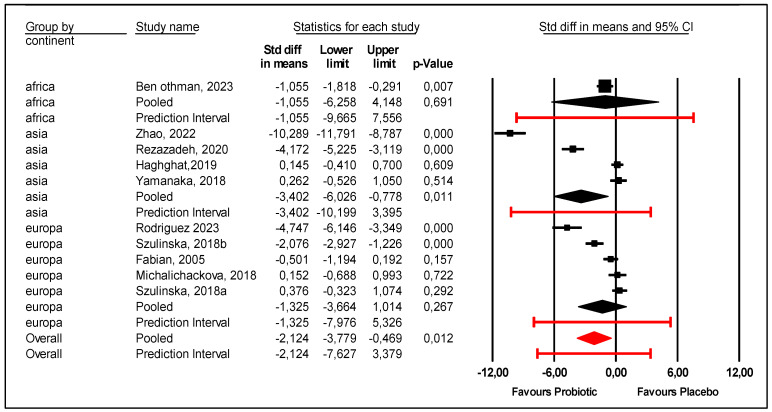
Forest Plot of the subgroup analysis according to country [9,21,22,23,24,25,26,27,28].

**Figure 5:**
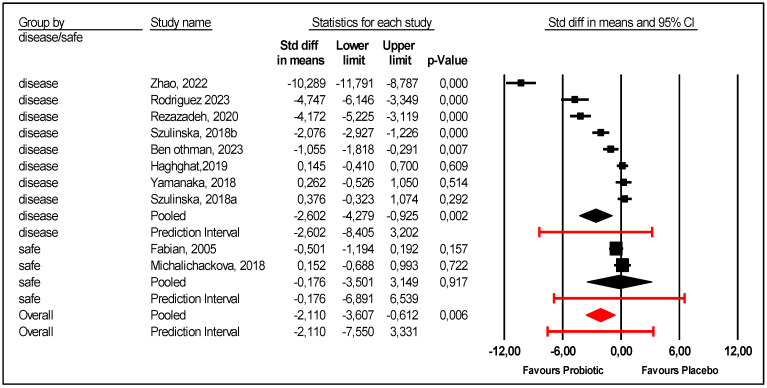
Forest plot of the subgroup analysis according to patients’ health status at inclusion [9,21,22,23,24,25,26,27,28].

**Figure 6:**
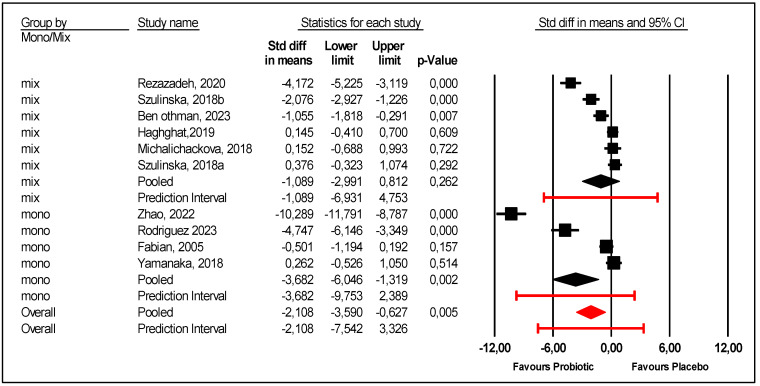
Forest plot of the subgroup analysis according to mono or multiple strains [9,21,22,23,24,25,26,27,28].

**Figure 7:**
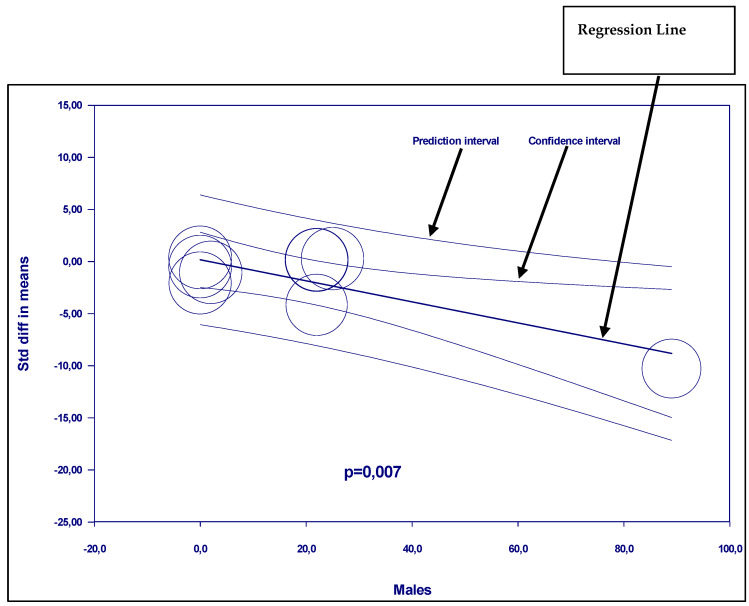
Regression of the subgroup analysis according to gender.

- An explanation of this heterogeneity is presented as follows.
- Regarding the continent, the subgroup of Asian patients were more likely to experience a reduction in uric acid levels with probiotics compared with placebo. The mean effect size was −3.402 with 95% CI (−6.026 to −0.778) (*p* = 0.011) (Figure 4). However, the prediction interval was large (−10.199 to +3.395), and heterogeneity among Asian patients was not explained. On the other hand, probiotics were not efficient for African (*p* = 0.691) or European patients (*p* = 0.267).

- Patients health status at inclusion was considered. There were two studies that included healthy adults: elite athletes and young healthy women [23,28]. In these two studies, there was no difference between the probiotic and placebo groups. In the subgroup of diseased patients, included patients had obesity [21,26], metabolic syndrome [22], hemodialysis [24], and HUA and/or gout [9,25,27]. Probiotics were more efficient for diseased patients than placebo (Figure 5); the mean effect size was −2.602 with 95% CI (−4.279 to −0.925) (*p* = 0.002). However, there was still unexplained heterogeneity: 95% PI (−8.405 to +3.202) for this subgroup of diseased patients (Figure 5).

- Single strains were assessed versus mixed multiple stains. Several strains were used with different doses. Mono-strain probiotics significantly decreased uric acid compared with placebo; mean effect size −3.682 with 95% CI (−6.046 to −1.319) (*p* = 0.002). However, there was an unexplained heterogeneity. In contrast, multi-strains probiotics were not efficient (Figure 6).
- Regarding gender, this meta-regression indicates that men are more sensitive to the effect of probiotics (Figure 7). There was a statically significant association between the proportion of male participants and the mean effect size (*p* = 0.007).

- Regarding age, BMI, and median follow-up, the meta-regression showed no statistical significance: age (*p* = 0.954), BMI (*p* = 0.73), median follow-up (*p* = 0.775).

## 4. Discussion

This meta-analysis demonstrated that probiotics reduced uric acid levels in Asian males who had disease and were treated with monostrain probiotics. In other words, this meta-analysis of 394 patients found a significantly statistical decrease in uric acid (UA) in the probiotic group. However, there was still a substantial heterogeneity within the subgroup analyses, suggesting considerable variability in the magnitude of the effect. This suggests that PBs can lower UA. PBs should be indicated in the curative management of HUA. However, substantial heterogeneity was observed across the included studies, suggesting considerable variability in the magnitude of the effect.

PBs have emerged as a promising approach for managing HUA and its associated conditions, such as gout [10]. Recent studies indicate that specific PB strains can modulate urate metabolism, reduce serum UA levels, and alleviate inflammatory responses [29,30]. PBs enhance the expression of UA transporters and promote intestinal excretion of UA, thereby lowering serum levels, and they reduce inflammatory markers such as IL-1β and LPS, which are associated with HUA [29,30]. PBs offer safety and efficacy in patients with HUA and gout [31]. Conventional treatments of these diseases include allopurinol or febuxostat as an alternative for allopurinol [32]. These urate-lowering treatments have many important side effects. The most frequently reported adverse effects of allopurinol include gastrointestinal disturbances, skin reactions, and liver dysfunction [33,34]. In rare cases, allopurinol can cause life-threatening conditions such as drug reaction with eosinophilia and systemic symptoms (DRESS) syndrome, Stevens–Johnson syndrome, and toxic epidermal necrolysis [35,36].

Our meta-analysis on the effects of PB supplementation on plasma UA levels revealed that men experienced significantly greater reductions compared with women. This indicates a potentially stronger therapeutic benefit of PBs on UA metabolism in males. Biological and physiological factors may underlie this difference, including sex-related variations in baseline UA levels, hormonal regulation, gut microbiota composition, and renal excretion capacity [37]. Studies have shown that men generally have higher baseline UA levels, which may influence their response to PB treatment [38]. Studies have shown that interventions targeting UA metabolism tend to have a more pronounced effect in individuals with higher initial UA levels, which could explain why men show greater reductions [39]. Sex-specific differences, particularly the influence of female sex hormones such as estrogen, may significantly affect the expression and/or activity of UA transporters like ABCG2 and GLUT9 (SLC2A9), thereby modulating the efficacy of PB interventions in UA clearance [40]. Men, who have lower renal UA clearance, may benefit more from PBs that stimulate gut excretion as an alternative pathway [41].

Regarding ethnicity, our meta-analysis stratified by ethnicity showed that studies involving Asian populations reported more pronounced and statistically significant reductions in UA levels than those focusing on other ethnic groups. This is possibly due to genetic predispositions, distinct gut microbiota profiles, or cultural dietary patterns. Chakrabarti et al. indicate that genetic factors and dietary habits can significantly influence metabolic responses to PBs [42]. Asian populations have a higher prevalence of genetic polymorphisms in URAT1 (SLC22A12) and GLUT9 (SLC2A9), key transporters involved in UA excretion [43]. These polymorphisms contribute to lower renal clearance of UA, making them more prone to HUA. Asians may tend to have higher UA levels than other populations, which could lead to a more pronounced effect when treated with PBs. Many Asian diets include fermented foods that naturally contain PB strains [44,45]. This habitual exposure might enhance the gut’s ability to process PBs efficiently. Compared with Western diets, Asian diets are often lower in purine-rich foods like processed red meat and sugars, which could amplify the impact of PBs on UA reduction.

Diseased participants with pre-existing HUA and/or diseases (obesity, metabolic syndrome, hemodialysis, HUA and/or gout) demonstrated more marked decreases in UA levels following PB intervention than their healthy counterparts [23,28]. Patients with HUA often exhibit gut dysbiosis, characterized by a reduction in uricolytic bacteria and an increase in pro-inflammatory species. PBs may have a greater effect on these individuals by restoring microbial balance, enhancing the activity of uricolytic bacteria, and promoting UA excretion [46,47].

Furthermore, in patients undergoing hemodialysis, PBs were associated with more substantial reductions in UA levels compared with non-dialyzed individuals. This observation points to a heightened PB effect in individuals with end-stage renal disease (ESRD). Potential mechanisms include compromised renal UA clearance, gut dysbiosis specific to ESRD, increased inflammatory burden, and altered gut–kidney axis dynamics in this population. PBs may provide an alternative route for UA elimination, particularly via the gut–liver–kidney axis, where intestinal microbes degrade and excrete UA, compensating for reduced renal clearance. This compensatory mechanism could explain why hemodialysis patients show more significant UA reductions when treated with PBs. Hemodialysis patients frequently exhibit severe gut dysbiosis, characterized by a decrease in beneficial bacteria such as Lactobacillus and Bifidobacterium and an increase in uremic toxin-producing bacteria such as *Escherichia coli* and *Clostridium* spp. [48,49]. PBs may reduce uremic toxins, leading to an indirect improvement in UA metabolism [50]. The results of our meta-analysis suggest that hemodialysis patients experience greater reductions in UA levels following PB treatment compared with non-dialyzed individuals. This is likely to be due to reduced renal UA excretion, severe gut dysbiosis, chronic inflammation, and enhanced gut–kidney axis interactions. These findings highlight the potential role of PBs as an adjunctive therapy in hemodialysis patients to improve metabolic outcomes. Further studies are needed to optimize strain selection, dosing, and long-term effects in this population.

As concerns mono versus multi-strain, our analysis also revealed that interventions using a mono-strain PB were more likely to yield statistically significant results than those using multi-strain combinations. This implies that mono-strain formulations might exert a more targeted effect on UA reduction [51]. Possible explanations include strain-specific bioactivity, inter-strain microbial competition, and variations in host response depending on the PB profile. PB effects are often strain-dependent; in fact, certain strains may have a more potent effect on UA metabolism than others [12,52,53,54,55,56]. When a mix of PBs is used, some less effective strains may dilute the effect of the more beneficial ones [57,58]. Specific bacterial strains, such as Lactobacillus plantarum or Bifidobacterium longum, have been identified as highly efficient uricolytics, capable of breaking down purines and promoting UA excretion [59]. Some PBs produce bacteriocins or metabolites that inhibit the growth or activity of co-administered strains, potentially reducing their UA-lowering effects. Different PB strains require different nutrients and ecological niches, leading to competition for resources in the gut. This could prevent some strains from colonizing effectively and exerting their full therapeutic effects [60]. Some PB strains exhibit non-linear dose responses, meaning that their effectiveness may not increase proportionally when combined with other strains [61].

In our meta-analysis, age did not affect the effect of PBs on uric acid levels. In a study involving elderly patients with chronic kidney disease, pre and probiotics did not significantly reduce serum uric acid levels, suggesting that age and underlying health conditions might influence the effectiveness of probiotics on uric acid metabolism [62]. As individuals age, their gut microbiome undergoes changes that can lead to dysbiosis, characterized by a decrease in beneficial bacteria and an increase in pro-inflammatory species [63,64]. Probiotics could potentially restore beneficial bacteria and modulate inflammatory responses in the elderly, improving conditions related to elevated uric acid levels [53,63]. However, the variability in gut microbiota among individuals underscores the complexity of developing age-specific probiotic treatments and necessitates further research to explore the nuanced interactions between age, gut health, and metabolic disorders [53]. Moreover, the chronic elevation of reactive oxygen species (ROS) in aging can exacerbate dysbiosis leading to systemic inflammation and metabolic dysfunction [63,65]. Such inflammatory states are hypothesized to impair the efficacy of probiotic interventions aimed at regulating uric acid levels, as seen in studies highlighting the relationship between inflammatory markers and gut microbiome health [66].

BMI did not impact PB’s effect, according to our meta-analysis. In the literature, while some studies report significant reductions in uric acid levels among individuals with higher BMI following probiotic supplementation [67], others present mixed results, necessitating further investigation to clarify these discrepancies [68].

Median follow-up did not influence PB’s effect, according to our meta-analysis. Several studies indicate that prolonged administration of probiotics can significantly reduce serum uric acid levels. A study involving *Lactobacillus rhamnosus* UA260 and *Lactobacillus plantarum* YU28 showed a substantial decrease in uric acid levels after two months of daily gavage at 10 9 CFU/day [52]. Similarly, a two-month trial with probiotic yogurt containing *Limosilactobacillus fermentum* GR-3 demonstrated a significant reduction in uric acid levels compared with conventional yogurt [9]. Another study found that *Lactobacillus brevis* MJM60390 reduced uric acid levels to normal within two weeks [69]. A six-month trial with a probiotic formulation showed a decrease in uric acid levels in patients with chronic kidney disease [70]. Additionally, a two-month trial with Pro-bio-X alongside febuxostat significantly decreased serum uric acid levels and reduced the rate of acute gout attacks [71]. A study involving the administration of a specific probiotic supplement daily for 12 weeks indicated substantial reductions in serum UA levels [26]. Another investigation into the effects of probiotic yogurt consumption for 8 weeks found a significant decrease in serum UA levels compared with regular yogurt [22]. The observed benefits of prolonged probiotic administration may be attributed to the ability of certain strains such as *Lactobacillus salivarius* CECT 30632 to efficiently metabolize purine-related metabolites, thereby reducing UA levels over time [25]. Optimal dosage and duration for probiotic supplementation remain uncertain, necessitating further research to establish standardized treatment protocols.

Several limitations should be acknowledged in this meta-analysis. First, the studies included a relatively small number of patients with diverse underlying pathologies and highly heterogeneous probiotic strains. The relatively small sample size may have reduced the statistical power of our analysis. We included eight RCTs and one CCT [21] only because trials with this design would provide the best evidence to answer our clinical question related to the efficacy of the investigated interventions. These conditions could have contributed to bias selection. To overcome this deficiency, the retained studies were rigorously assessed and scored using the methodological index of non-randomized studies (MINORS) and JADAD. It remained impossible to match all patient groups and to avoid heterogeneity.

Further RCTs comparing probiotics to placebo are needed to explore the sources of this variability and identify specific patient populations or treatment characteristics that might further influence the effectiveness of PBs.

## Figures and Tables

**Table 1:** Characteristics of included studies.

First Author	Country of Origin	Year of Publication	Type of Disease	Number of Patients (Probiotics/ Comparator)	Sex Ratio (M/W)	Mean Ages (Years)	Mean BMI (kg/m^2^)	Intervention	Dosage	Comparator	Follow-Up (Months)	Uric Acid Before Intervention (mg/dL)	Uric Acid After Intervention (mg/dL)	Method of Uric Acid Dosage	JADAD /5 or MINORS/24
Ben Othman [21]	Africa	2023	Obesity	30 (15/15)	-	49	-	one tab of: *B. longum* *L. helveticus Lactococcal latis* *S. thermophilus*	10 × 10^9^ UFC/capsule	Low-calorie diet	1	4.97 ± 1.7	4.78 ± 1.34	NA	18 *
Haghighat [24]	Asia	2019	Hemodialysis	50 (25/25)	1.2	47	23	5 g of probiotic powder: *B. bifidum* *B. lactis* *B. longum*	2.7 × 10^7^ CFU/g each	Placebo: 20 g of maltodextrin powder	3	6.99 ± 2.06	6.98 ± 1.81	Clauss technique and the uricase enzymatic test	5
Michalichova [23]	Europa	2018	Elite athletes	22 (10/12)	22	22	23	1 capsule of *L. helveticus*	2 × 10^10^	Placebo	3.5	4.02 ± 0.77	3.95 ± 1.41	NA	4
Rezazadeh [22]	Asia	2020	Metabolic syndrome	44 (22/22)	1	44	32	300 g of yogurt containing *L. bulgaricus*, *S. thermophile*, *B. lactis*, *L. acidophilus*	3.55 × 10^6^ CFU/g of *B. lactis*, 4.41 × 10^6^ CFU/g of *L. acidophilus*/d	Conventional yogurt: *L. bulgaricus*, *S. thermophile*	2	6.33 ± 1.4	4.65 ± 0.72	enzymatic method	5
Rodriguez [25]	Europa	2023	Hyperuricemia (>7 mg/dL), a history of recurrent gout episodes (≥3 episodes/year)	30 (15/15)	-	54	32	*L. Salivarus* *CECT 30632*	10^10^ CFU	Allopurinol	6	9.04 ± 0.25	9.03 ± 0.25	HPLC	5
Szulinska (a) [26]	Europa	2018	Obese post-menopausal	36 (24/12)	0	57	36	Sachets containing 2 g of probiotic divided in 2 equal doses: *B. bifidum* *B. lactis w51* *L. acidophilus* *L. brevis* *L. casei* *L. salivarius* *L. lactis w19* *L. lactis w58*	1 × 10^10^ CFU/d	Placebo	3	5.26 ± 1.04	5.28 ± 1.09	Dimension EXL with LM Integrated Chemistry System Analyzer	5
Szulinska (b) [26]	Europa	2018	Obese post-menopausal	35 (23/12)	0	58	36		2.5 × 10^9^ CFU/d	Placebo	3	6.02 ± 0.71	5.35 ± 0.91		5
Yamanaka [27]	Asia	2018	Hyperuricemia and/or gout	17 (9/8)	17	63	25	100 g of yogurt with *L. bulgaricus*, *S. thermophile*, *L. delbruecki*	8.5 × 10^7^ CFU/g	Conventional yogurt: *L. bulgaricus*, *S. thermophile*	2	8.7 ± 1	8.7 ± 1.2	Uricase-POD method	3
Zhao [9]	Asia	2022	UA > 7 mg/dL	97 (52/45)	2.87	39	28.6	Yogurt containing *L. bulgaricus*, *S. thermophiles*, *Limosi L. fermentum GR-3*	2.0 × 10^9^ CFU/g of each bacterial strain/d	Conventional yogurt: *L. bulgaricus*, *S. thermophile*	2	9.65 ± 0.76	7.12 ± 0.22	NA	5
Fabian [28]	Europa	2007	Healthy women	33 (17/16)	0/33	24	21	Yogurt containing *L. bulgaricus*, *S. thermophiles*, *L. paracasei* subsp. *Paracasei*	3.6 × 10^8^ CFU/g	Conventional yogurt: *L. bulgaricus*, *S. thermophile*	1	2.45 ± 0.42	2.2 ± 0.48	Enzymatic method	5

BMI: body mass index. B = *bifidobacterium*; L = *lactobacillus*; UFC = colony-forming unit. * The only CCT study included and evaluated by MINORS [9,21,22,23,24,25,26,27,28].

## Abbreviations

The following abbreviations are used in this manuscript:

RCT	Randomized controlled trial
PRISMA	Preferred Reporting Items for Systematic Reviews and Meta-Anlyses
HUA	Hyperuricemia
CVD	Cardiovascular disease
USA	United States of America
CKD	Chronic kidney disease
XOD	Xanthine oxidase
PB	Probiotic
CCT	Controlled clinical trial
BMI	Body mass index
IQR	Interquartile range
SD	Standard deviation
PI	Prediction interval
CI	Confidence interval
IL1β	Interleukin-1 beta
LPS	Lipopolysaccharide
ESRD	End-stage renal disease
CFU	Colony-forming unit

## References

[1] Chen-Xu M., Yokose C., Rai S.K., Pillinger M.H., Choi H.K. (2019). Contemporary Prevalence of Gout and Hyperuricemia in the United States and Decadal Trends: The National Health and Nutrition Examination Survey, 2007–2016. Arthritis Rheumatol..

[2] Jiang J., Zhang T., Liu Y., Chang Q., Zhao Y., Guo C., Xia Y. (2023). Prevalence of Diabetes in Patients with Hyperuricemia and Gout: A Systematic Review and Meta-Analysis. Curr. Diabetes Rep..

[3] Schwotzer N., Auberson M., Livio F., So A., Bonny O. (2022). Hyperuricémie et maladie rénale: Prise en charge. Rev. Med. Suisse.

[4] Kojima S., Matsui K., Hiramitsu S., Hisatome I., Waki M., Uchiyama K., Yokota N., Tokutake E., Wakasa Y., Jinnouchi H. (2019). Febuxostat for cerebral and cardiorenovascular events prevention study. Eur. Heart J..

[5] Yokota T., Fukushima A., Kinugawa S., Okumura T., Murohara T., Tsutsui H. (2018). Randomized trial of effect of urate-lowering agent febuxostat in chronic heart failure patients with hyperuricemia (LEAF-CHF) study design. Int. Heart J..

[6] Mahomoodally M.F., Coodian K., Hosenally M., Zengin G., Shariati M.A., Abdalla A.N., Alhazmi H.A., Khuwaja G., Mohan S., Khalid A. (2024). Herbal remedies in the management of hyperuricemia and gout: A review of in vitro, in vivo and clinical evidences. Phytother. Res..

[7] Singh J.A., Cleveland J.D. (2020). Hypersensitivity reactions with allopurinol and febuxostat: A study using the Medicare claims data. Ann. Rheum. Dis..

[8] Roddy E., Bajpai R., Forrester H., Partington R.J., Mallen C.D., Clarson L.E., Padmanabhan N., Whittle R., Muller S. (2023). Safety of colchicine and NSAID prophylaxis when initiating urate-lowering therapy for gout: Propensity score-matched cohort studies in the UK Clinical Practice Research Datalink. Ann. Rheum. Dis..

[9] Zhao S., Feng P., Hu X., Cao W., Liu P., Han H., Jin W., Li X. (2022). Probiotic *Limosilactobacillus fermentum* GR-3 ameliorates human hyperuricemia via degrading and promoting excretion of uric acid. iScience.

[10] Zhang X., Jiang J., Xin J., Sun N., Zhao Z., Gan B., Jiang Y., Gong X., Li H., Ma H. (2024). Preventive effect of *Lactobacillus johnsonii* YH1136 against uric acid accumulation and renal damages. Front. Microbiol..

[11] Chen T., Qiu S. (2024). Recent Status of Probiotics in the Prevention and Treatment of Hyperuricemia (HUA). MedScien.

[12] Lin J.-H., Lin C.-H., Kuo Y.-W., Liao C.-A., Chen J.-F., Tsai S.-Y., Li C.-M., Hsu Y.-C., Huang Y.-Y., Hsia K.-C. (2024). Probiotic *Lactobacillus fermentum* TSF331, *Lactobacillus reuteri* TSR332, and *Lactobacillus plantarum* TSP05 improved liver function and uric acid management-A pilot study. PLoS ONE.

[13] Page M.J., Moher D., Bossuyt P.M., Boutron I., Hoffmann T.C., Mulrow C.D., Shamseer L., Tetzlaff J.M., Akl E.A., Brennan S.E. (2021). PRISMA 2020 explanation and elaboration: Updated guidance and exemplars for reporting systematic reviews. BMJ.

[14] Slim K., Nini E., Forestier D., Kwiatkowski F., Panis Y., Chipponi J. (2003). Methodological index for non-randomized studies (minors): Development and validation of a new instrument. ANZ J. Surg..

[15] Obtaining standard deviations from standard errors and confidence intervals for group means. https://handbook-5-1.cochrane.org/chapter_7/7_7_3_2_obtaining_standard_deviations_from_standard_errors_and.htm.

[16] Borenstein M., Hedges L.V., Higgins J.P.T., Rothstein H.R. (2021). Prediction Intervals. Introduction to Meta-Analysis.

[17] Borenstein M. (2019). Common Mistakes in Meta-Analysis and How to Avoid Them, the Prediction Interval.

[18] Dziri C., Fingerhut A. (2025). Up-to-date composition and critical appraisal of meta-analyses of comparative studies. Ann. Laparosc. Endosc. Surg..

[19] Thompson S.G., Higgins J.P.T. (2002). How should meta-regression analyses be undertaken and interpreted?. Stat. Med..

[20] Borenstein M., Hedges L.V., Higgins J.P.T., Rothstein H.R. (2005). Comprehensive Meta-Analysis.

[21] Ben Othman R., Ben Amor N., Mahjoub F., Berriche O., El Ghali C., Gamoudi A., Jamoussi H. (2023). A clinical trial about effects of prebiotic and probiotic supplementation on weight loss, psychological profile and metabolic parameters in obese subjects. Endocrinol. Diabetes Metab..

[22] Rezazadeh L., Alipour B., Jafarabadi M.A., Behrooz M., Gargari B.P. (2021). Daily consumption effects of probiotic yogurt containing *Lactobacillus acidophilus* La5 and Bifidobacterium lactis Bb12 on oxidative stress in metabolic syndrome patients. Clin. Nutr. ESPEN.

[23] Michalickova D., Kotur-Stevuljevic J., Miljkovic M., Dikic N., Kostic-Vucicevic M., Andjelkovic M., Koricanac V., Djordjevic B. (2018). Effects of Probiotic Supplementation on Selected Parameters of Blood Prooxidant-Antioxidant Balance in Elite Athletes: A Double-Blind Randomized Placebo-Controlled Study. J. Hum. Kinet..

[24] Haghighat N., Mohammadshahi M., Shayanpour S., Haghighizadeh M.H. (2019). Effect of Synbiotic and Probiotic Supplementation on Serum Levels of Endothelial Cell Adhesion Molecules in Hemodialysis Patients: A Randomized Control Study. Probiotics Antimicrob. Proteins.

[25] Rodríguez J.M., Garranzo M., Segura J., Orgaz B., Arroyo R., Alba C., Beltrán D., Fernández L. (2023). A randomized pilot trial assessing the reduction of gout episodes in hyperuricemic patients by oral administration of *Ligilactobacillus salivarius* CECT 30632, a strain with the ability to degrade purines. Front. Microbiol..

[26] Szulińska M., Łoniewski I., van Hemert S., Sobieska M., Bogdański P. (2018). Dose-Dependent Effects of Multispecies Probiotic Supplementation on the Lipopolysaccharide (LPS) Level and Cardiometabolic Profile in Obese Postmenopausal Women: A 12-Week Randomized Clinical Trial. Nutrients.

[27] Yamanaka H., Taniguchi A., Tsuboi H., Kano H., Asami Y. (2019). Hypouricaemic effects of yoghurt containing *Lactobacillus gasseri* PA-3 in patients with hyperuricaemia and/or gout: A randomised, double-blind, placebo-controlled study. Mod. Rheumatol..

[28] Fabian E., Elmadfa I. (2007). The effect of daily consumption of probiotic and conventional yoghurt on oxidant and anti-oxidant parameters in plasma of young healthy women. Int. J. Vitam. Nutr. Res..

[29] Cao J., Wang T., Liu Y., Zhou W., Hao H., Liu Q., Yin B., Yi H. (2023). *Lactobacillus fermentum* F40-4 ameliorates hyperuricemia by modulating the gut microbiota and alleviating inflammation in mice. Food Funct..

[30] Li Y., Zhu J., Lin G., Gao K., Yu Y., Chen S., Chen L., Chen Z., Li L. (2022). Probiotic effects of Lacticaseibacillus rhamnosus 1155 and Limosilactobacillus fermentum 2644 on hyperuricemic rats. Front. Nutr..

[31] Zeng L., Deng Y., He Q., Yang K., Li J., Xiang W., Liu H., Zhu X., Chen H. (2022). Safety and efficacy of probiotic supplementation in 8 types of inflammatory arthritis: A systematic review and meta-analysis of 34 randomized controlled trials. Front. Immunol..

[32] Peng X., Li X., Xie B., Lai Y., Sosnik A., Boucetta H., Chen Z., He W. (2023). Gout therapeutics and drug delivery. J. Control. Release.

[33] Begg A. (2023). Allopurinol. Pract. Diabetes.

[34] McInnes G.T., Lawson D.H., Jick H. (1981). Acute adverse reactions attributed to allopurinol in hospitalised patients. Ann. Rheum. Dis..

[35] Calin A. (1978). Allopurinol Toxicity Masquerading as Malignancy. JAMA.

[36] Fagugli R.M., Gentile G., Ferrara G., Brugnano R. (2008). Acute renal and hepatic failure associated with allopurinol treatment. Clin. Nephrol..

[37] Razavi A.C., Potts K.S., Kelly T.N., Bazzano L.A. (2019). Sex, gut microbiome, and cardiovascular disease risk. Biol. Sex. Differ..

[38] Wang Y., Li S., Li X., Wang M., Huang B., Feng K., Cui J. (2025). Association between prebiotic, probiotic consumption and hyperuricemia in U.S. adults: A cross-sectional study from NHANES 2011-2018. Front. Nutr..

[39] Cicero A.F.G., Fogacci F., Cincione R.I., Tocci G., Borghi C. (2021). Clinical Effects of Xanthine Oxidase Inhibitors in Hyperuricemic Patients. Med. Princ. Pract..

[40] Halperin Kuhns V.L., Woodward O.M. (2020). Sex Differences in Urate Handling. Int. J. Mol. Sci..

[41] Sumino H., Ichikawa S., Kanda T., Nakamura T., Sakamaki T. (1999). Reduction of serum uric acid by hormone replacement therapy in postmenopausal women with hyperuricaemia. Lancet.

[42] Chakrabarti A., Geurts L., Hoyles L., Iozzo P., Kraneveld A.D., La Fata G., Miani M., Patterson E., Pot B., Shortt C. (2022). The microbiota-gut-brain axis: Pathways to better brain health. Perspectives on what we know, what we need to investigate and how to put knowledge into practice. Cell Mol. Life Sci. CMLS.

[43] Ichida K. (2024). Uric Acid Metabolism, Uric Acid Transporters and Dysuricemia. Yakugaku Zasshi.

[44] Rhee S.J., Lee J.-E., Lee C.-H. (2011). Importance of lactic acid bacteria in Asian fermented foods. Microb. Cell Factories.

[45] Swain M.R., Anandharaj M., Ray R.C., Rani R.P. (2014). Fermented fruits and vegetables of Asia: A potential source of probiotics. Biotechnol. Res. Int..

[46] Cooper T.E., Khalid R., Chan S., Craig J.C., Hawley C.M., Howell M., Johnson D.W., Jaure A., Teixeira-Pinto A., Wong G. (2023). Synbiotics, prebiotics and probiotics for people with chronic kidney disease—Cooper, TE—2023|Cochrane Library. Cochrane Database Syst. Rev..

[47] Nourizadeh R., Sepehri B., Abbasi A., Sayyed R.Z., Khalili L., Sayyed R.Z., Khan M. (2022). Impact of Probiotics in Modulation of Gut Microbiome. Microbiome-Gut-Brain Axis Implic. Health.

[48] Wang J., Chen Y., Zhong H., Chen F., Regenstein J., Hu X., Cai L., Feng F. (2022). The gut microbiota as a target to control hyperuricemia pathogenesis: Potential mechanisms and therapeutic strategies. Crit. Rev. Food Sci. Nutr..

[49] Lim X., Ooi L., Ding U., Wu H.H.L., Chinnadurai R. (2024). Gut Microbiota in Patients Receiving Dialysis: A Review. Pathogens.

[50] Kuznetzova A.B., Prazdnova E.V., Chistyakov V.A., Kutsevalova O.Y., Batiushin M.M. (2022). Are Probiotics Needed In Nephrology?. Nephrol. St.-Petersburg.

[51] Prasad C., Iqbal U., Westfall S., Prakash S. (2017). Management of hyperuricemia and gout by prebiotics and probiotics: Potentials and limitations. Int. J. Probiotics Prebiotics.

[52] Wang Q., Liang J., Zou Q., Wang W., Yan G., Guo R., Yuan T., Wang Y., Liu X., Liu Z. (2024). Tryptophan Metabolism-Regulating Probiotics Alleviate Hyperuricemia by Protecting the Gut Barrier Integrity and Enhancing Colonic Uric Acid Excretion. J. Agric. Food Chem..

[53] Liang L., Meng Z., Zhang F., Jianguo Z., Fang S., Hu Q., Tang X., Li Y. (2023). *Lactobacillus gasseri* LG08 and *Leuconostoc mesenteroides* LM58 exert preventive effect on the development of hyperuricemia by repairing antioxidant system and intestinal flora balance. Front. Microbiol..

[54] Wang Z., Huang Y., Yang T., Song L., Xiao Y., Chen Y., Chen M., Li M., Ren Z. (2024). *Lactococcus cremoris* D2022 alleviates hyperuricemia and suppresses renal inflammation via potential gut-kidney axis. Food Funct..

[55] Wu J., Aga L., Tang L., Li H., Wang N., Yang L., Zhang N., Wang X., Wang X. (2024). *Lacticaseibacillus paracasei* JS-3 Isolated from “Jiangshui” Ameliorates Hyperuricemia by Regulating Gut Microbiota and iTS Metabolism. Foods.

[56] Hussain A., Rui B., Ullah H., Dai P., Ahmad K., Yuan J., Liu Y., Li M. (2024). *Limosilactobacillus reuteri* HCS02-001 Attenuates Hyperuricemia through Gut Microbiota-Dependent Regulation of Uric Acid Biosynthesis and Excretion. Microorganisms.

[57] Chapman C.M.C., Gibson G.R., Rowland I. (2012). In vitro evaluation of single- and multi-strain probiotics: Inter-species inhibition between probiotic strains, and inhibition of pathogens. Anaerobe.

[58] Buiatte V., Schultheis M., Lorenzoni A.G. (2023). Deconstruction of a multi-strain Bacillus-based probiotic used for poultry: An in vitro assessment of its individual components against C. perfringens. BMC Res. Notes.

[59] Fu Y., Luo X.-D., Li J.-Z., Mo Q.-Y., Wang X., Zhao Y., Zhang Y.-M., Luo H.-T., Xia D.-Y., Ma W.-Q. (2024). Host-derived *Lactobacillus plantarum* alleviates hyperuricemia by improving gut microbial community and hydrolase-mediated degradation of purine nucleosides. eLife.

[60] Sanders M.E., Benson A., Lebeer S., Merenstein D.J., Klaenhammer T.R. (2018). Shared mechanisms among probiotic taxa: Implications for general probiotic claims. Curr. Opin. Biotechnol..

[61] Forssten S., Ouwehand A.C. (2020). Dose-Response Recovery of Probiotic Strains in Simulated Gastro-Intestinal Passage. Microorganisms.

[62] Pavan M. (2016). Influence of prebiotic and probiotic supplementation on the progression of chronic kidney disease. Minerva Urol. Nephrol..

[63] Buford T.W. (2017). (Dis)Trust your gut: The gut microbiome in age-related inflammation, health, and disease. Microbiome.

[64] Wang Z., Li Y., Liao W., Huang J., Liu Y., Li Z., Tang J. (2022). Gut microbiota remodeling: A promising therapeutic strategy to confront hyperuricemia and gout. Front. Cell. Infect. Microbiol..

[65] Shi W., Cai Z., Ren X., Wang J., Zhou H., Chen Z. (2025). The relationship between serum uric acid and accelerated aging in middle-aged and older adults: A prospective cohort study based on CHARLS. J. Nutr. Health Aging.

[66] Chenhuichen C., Cabello-Olmo M., Barajas M., Izquierdo M., Ramírez-Vélez R., Zambom-Ferraresi F., Martínez-Velilla N. (2022). Impact of probiotics and prebiotics in the modulation of the major events of the aging process: A systematic review of randomized controlled trials. Exp. Gerontol..

[67] Rasaei N., Heidari M., Esmaeili F., Khosravi S., Baeeri M., Tabatabaei-Malazy O., Emamgholipour S. (2024). The effects of prebiotic, probiotic or synbiotic supplementation on overweight/obesity indicators: An umbrella review of the trials’ meta-analyses. Front. Endocrinol..

[68] Yarahmadi A., Afkhami H., Javadi A., Kashfi M. (2024). Understanding the complex function of gut microbiota: Its impact on the pathogenesis of obesity and beyond: A comprehensive review. Diabetol. Metab. Syndr..

[69] Lee Y., Kim N., Werlinger P., Suh D.-A., Lee H., Cho J.-H., Cheng J. (2022). Probiotic Characterization of *Lactobacillus brevis* MJM60390 and In Vivo Assessment of Its Antihyperuricemic Activity. J. Med. Food.

[70] Ranganathan N., Ranganathan P., Friedman E.A., Joseph A., Delano B., Goldfarb D.S., Tam P., Rao A.V., Anteyi E., Musso C.G. (2010). Pilot study of probiotic dietary supplementation for promoting healthy kidney function in patients with chronic kidney disease. Adv. Ther..

[71] Zhao F., Tie N., Kwok L.-Y., Ma T., Wang J., Man D., Yuan X., Li H., Pang L., Shi H. (2024). Baseline gut microbiome as a predictive biomarker of response to probiotic adjuvant treatment in gout management. Pharmacol. Res..

